# Torque-ratio-adjustable ankle-foot exoskeleton for resisting perturbation in forward direction within fan-shaped region of pelvis horizontal plane

**DOI:** 10.3389/fbioe.2024.1429605

**Published:** 2024-08-05

**Authors:** Yuyao Liu, Ronglei Sun, Kaijie Zou, Ying Li, Peng Xu

**Affiliations:** ^1^ Institute of Medical Equipment Science and Engineering, Huazhong University of Science and Technology, Wuhan, China; ^2^ State Key Laboratory of Intelligent Manufacturing Equipment and Technology, Huazhong University of Science and Technology, Wuhan, China; ^3^ School of Mechanical Science and Engineering, Huazhong University of Science and Technology, Wuhan, China

**Keywords:** ankle-foot exoskeleton, perturbed standing, perturbation direction, torque ratio, adjusting strategy, bioinspired design, subtalar joint, inversion

## Abstract

**Introduction:** The ankle-foot exoskeleton has been demonstrated to help users resist anterior perturbation in the horizontal pelvis plane. However, its effects on perturbations in other directions remain unclear. This paper focuses on how the ankle-foot exoskeleton helps people resist perturbations coming from forward directions within the fan-shaped region in the pelvis horizontal plane.

**Methods:** Firstly, we proposed and validated a hypothesis that the human torque ratio of inversion to plantar flexion torque would change with the perturbation directions of anterior (dir0) and 45° deviating from anterior to left (dir45). Subsequently, based on the regulation demand, we developed an ankle-foot exoskeleton that can adjust the torque ratio delivered to the human body by controlling the forces on two cross-arranged cables. Finally, we evaluated and compared the assistance performance of three powered assistive modes (NM, medBD, and latBD) with the unpowered one (UN) by setting different force pairs in two cables.

**Results:** The results showed that, with the assistance, the margin of stability was increased and the standard deviations of ankle-foot segmental movements were decreased. Meanwhile, the biological inversion torque has a significant difference among the three assistive modes. Compared to the UN, the latBD was shown to reduce the biological inversion torque by 15.8 % and 13.7 % in response to the dir0 and dir45 perturbations, respectively, while the reductions for the NM and medBD were smaller. It was also observed that the torque ratios, generated by the human and the exoskeleton in latBD mode, differed by about 0.1 under dir0 and 0.08 under dir45, while the physiologically similarity of the exoskeleton torque ratio in NM and medBD modes were smaller. Based on the above results, we found that the more physiologically similar the exoskeleton torque ratio, the better the assistive performance.

**Discussion:** The findings demonstrated that the torque-ratio-adjustable exoskeleton could support human resistance to perturbations coming from forward directions within a fan-shaped region in the pelvis horizontal plane and indicated that the exoskeleton’s torque ratio should be carefully modulated to match the ratio of the human under various environmental conditions for better assistive performance.

## 1 Introduction

It has been demonstrated that using an ankle-foot exoskeleton improves user forward balance in the sagittal plane (Asbeck et al., 2015; Emmens et al., 2018; Beck et al., 2023). Nonetheless, most perturbations do not happen entirely in the sagittal plane, usually along with coronal components. It is still unknown how to assist humans in accounting for perturbation in forward direction within fan-shaped region of pelvis horizontal plane with an ankle-foot exoskeleton.

There have been studies on human responses to multi-directional perturbations, showing that human responses differ depending on the direction of perturbation (Henry et al., 1998; Torres-Oviedo and Ting, 2007; Jones et al., 2008; Chvatal and Ting, 2013). The perturbation was delivered by the treadmill translation, and the direction of perturbation was determined by the position of the human standing on the treadmill. At the joint level, it was shown that the postural responses in the sagittal and coronal planes were not independent. Instead, the relative contribution of hip/trunk *versus* ankle strategies was redistributed due to the direction-specific biomechanical constraints at the ankle joint (Jones et al., 2008). At the muscle level, Horak’s team found that the medial gastrocnemius, soleus, and peroneus muscles of the left leg showed their maximum activation responding to the treadmill translation directions between the back and right (Henry et al., 1998). Using nonnegative matrix factorization, Ting’s team extracted muscle synergies characterizing human postural responses. The ankle strategy was consistent with the muscle synergy that activated the gastrocnemius, soleus, and peroneus. Symmetry to the work of Horak’s team, this synergy of the right leg was activated to respond to the treadmill translation directions between the back and left (Torres-Oviedo and Ting, 2007). In a subsequent study by Ting’s team, the muscle synergy corresponding to the ankle strategy was found to contribute to the backward ground reaction force and back-medial acceleration of the center of mass (Chvatal and Ting, 2013). To focus on the “ankle” strategy, the perturbation should be delivered by horizontal forward pulling on the pelvis (Robert et al., 2018). In this paper, the pulling direction was set to be standard forward and a non-standard forward instance: left-forward. Decomposing the horizontal perturbation into the sagittal and coronal planes, the forward component was mainly resisted by the plantar flexion torque of the talocrural joint, while the left component was mainly counteracted by the inversion torque of the left subtalar joint. Therefore, we inferred that if the perturbation direction changed, the needed torque ratio of inversion to plantar flexion would change, owing to the change in the ankle-foot muscle activation pattern.

The assistive torques of current ankle-foot exoskeletons were usually designed to mimic human torque profiles to minimize interference with the user’s normal movements (Sawicki and Ferris, 2008; Emmens et al., 2018; Nuckols et al., 2021; Pridham and Stirling, 2023). However, they only considered the assistance delivered to the talocrural joint, because the plantar flexion torque contributed most to the ambulation they focused on. Recently, a simulation study investigated how exoskeleton torques applied to the talocrural and subtalar joints altered the center of mass kinematics during walking and found that the type of exoskeleton could change the center of mass kinematics (Bianco et al., 2023). Although the torque magnitudes applied to the talocrural and subtalar joints for simulation were the same, the author argued that different combinations of torque magnitudes at these joints could facilitate precise control over the direction of velocity changes. Therefore, we supposed that the exoskeleton torque ratio of inversion to plantar flexion should be controlled to mimic the human ratio varying across perturbed conditions.

Most current ankle-foot exoskeleton structures pulled the back bottom of shoes across talocrural and subtalar joints to supply the assistive forces, mimicking the soleus muscle, which meant that these exoskeletons could deliver torques to both joints simultaneously (Bryan et al., 2021; Nuckols et al., 2021; Zhang and Collins, 2021; Chen et al., 2022; Schmitz et al., 2022; Wu et al., 2024). However, the positions of exoskeleton attachment points relative to the human body were fixed, leading to an uncontrollable exoskeleton torque ratio, which may hinder the exoskeleton from being more effective. Instead, exoskeleton structures that could actively assist two joints separately have the potential to realize the modulation of torque-ratio. Baek’s team proposed a two-degree-of-freedom ankle-foot exoskeleton (Choi et al., 2020; Choi et al., 2022). The axes of rotation of two hinge joints were designed by calculating the spatial formula of the talocrural joint and subtalar joint based on anatomical data. The talocrural joint and subtalar joint were actively controlled to rotate by two pneumatic artificial muscles, mimicking the soleus and peroneus longus. However, the above exoskeleton had a hinge joint. When providing active support, it can be challenging to maintain dynamic alignment between the exoskeleton’s hinge joint and the wearer’s talocrural joint due to the floating axis of the wearer’s joint. Soft exoskeletons can overcome this feedback. Park et al. have developed a bio-inspired tendon-ligament-skin architecture that can assist ankle-foot movement with a low profile (Park et al., 2014). The eversion was achieved by the artificial peroneus tertius, while the inversion was realized by the artificial tibialis anterior. By co-activating the agonist-antagonist artificial muscles, the combined architecture not only provided physical support by increasing the stiffness of the ankle joint but also created desired ankle motions. The proposed scheme provided a flexible combination of actuators to satisfy various requirements of ankle rehabilitation. However, two artificial muscles were attached to both sides of the midfoot to actively assist with inversion/eversion. These muscles also functioned as dorsiflexors, which could counteract part of the force from the plantar flexors and reduce the efficiency of plantar flexion. Humans have a more efficient muscle layout for movement. Some plantar flexors can also function as invertors and evertors (Neumann, 2016). Thus, it was still needed to develop a soft exoskeleton that could support not only the plantar flexion torque but also the inversion torque and modulate the torque ratio of inversion to plantar flexion.

In this paper, we first validated the hypothesis that the human torque ratio would change according to the perturbation direction. Then, we developed a torque-ratio-adjustable ankle-foot exoskeleton for resisting perturbation in forward direction within fan-shaped region of pelvis horizontal plane. Finally, we compared the torque-ratio difference between the exoskeleton and human among three assistive modes to validate the hypothesis that the smaller the difference, the better the assistive performance.

## 2 Materials and methods

### 2.1 Ankle-foot exoskeleton

Ankle-foot muscles are fixed on the bone and function uniquely because of their different attachments. The soleus (SOL), lateral gastrocnemius (LGAS), and medial gastrocnemius (MGAS) are the primary plantar flexors but also offer a small number of inversion torque. On the contrary, the tibialis posterior (TP) is the primary invertor but can only provide small plantar flexion torques. In addition, the peroneus longus (PERL) is the main evertor that can antagonize excessive inversion torque, while the tibialis anterior (TA) is the primary dorsiflexor that can counteract excessive plantar flexion torque. Although each of the muscles produces a fixed biological torque ratio of inversion to plantar flexion, the muscle group can change the torque ratio by activating different patterns.

**FIGURE 1:**
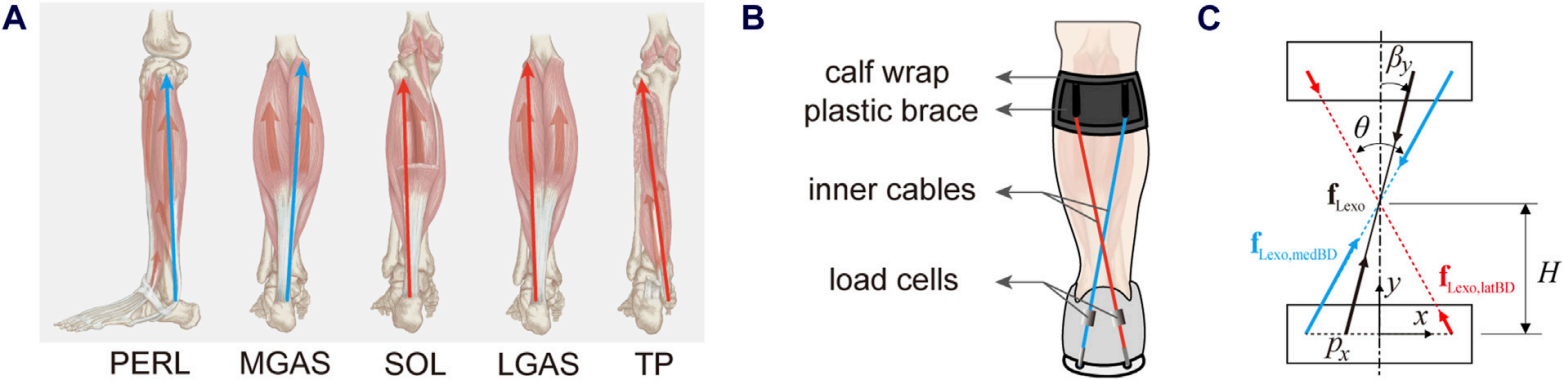
**(A)** The orientation of main ankle-foot plantar flexors, including the peroneus longus (PERL), the medial gastrocnemius (MGAS), the soleus (SOL), the lateral gastrocnemius (LGAS) and the tibialis posterior (TP). **(B)** The scheme of the torque-ratio-adjustable ankle-foot exoskeleton on the left leg. **(C)** The force orientation regulation mechanism of the left exoskeleton. The red represents muscles that lean to the lateral side of the body or insert into the medial side of the heel, while the blue represents muscles that lean to the medial side of the body or insert into the lateral side of the heel.

Most current exoskeletons have a structure with only one Bowden cable attached between the heel and tibia parallel to the triceps surae, thus they cannot modulate their torque ratios of inversion to plantar flexion torque. Inspired by the torque-ratio adaptation mechanism of the muscle group, if the exoskeleton has a group of cables with different orientations relative to the ankle-foot segment, it would be able to provide different torque functions. According to the orientations of ankle-foot muscles, we divided the muscles into two groups (Figure 1A). One containing SOL, LGAS, and TP leans to the lateral side of the body or is inserted into the medial side of the heel, while the other containing MGAS and PERL leans to the medial side of the body or insert into the lateral side of the heel. Our previous exoskeleton has two cables for each leg (Liu et al., 2023). Thus, we modified the directions of the two ropes behind the shank from parallel to decussate layout (Figure 1B).

**FIGURE 2:**
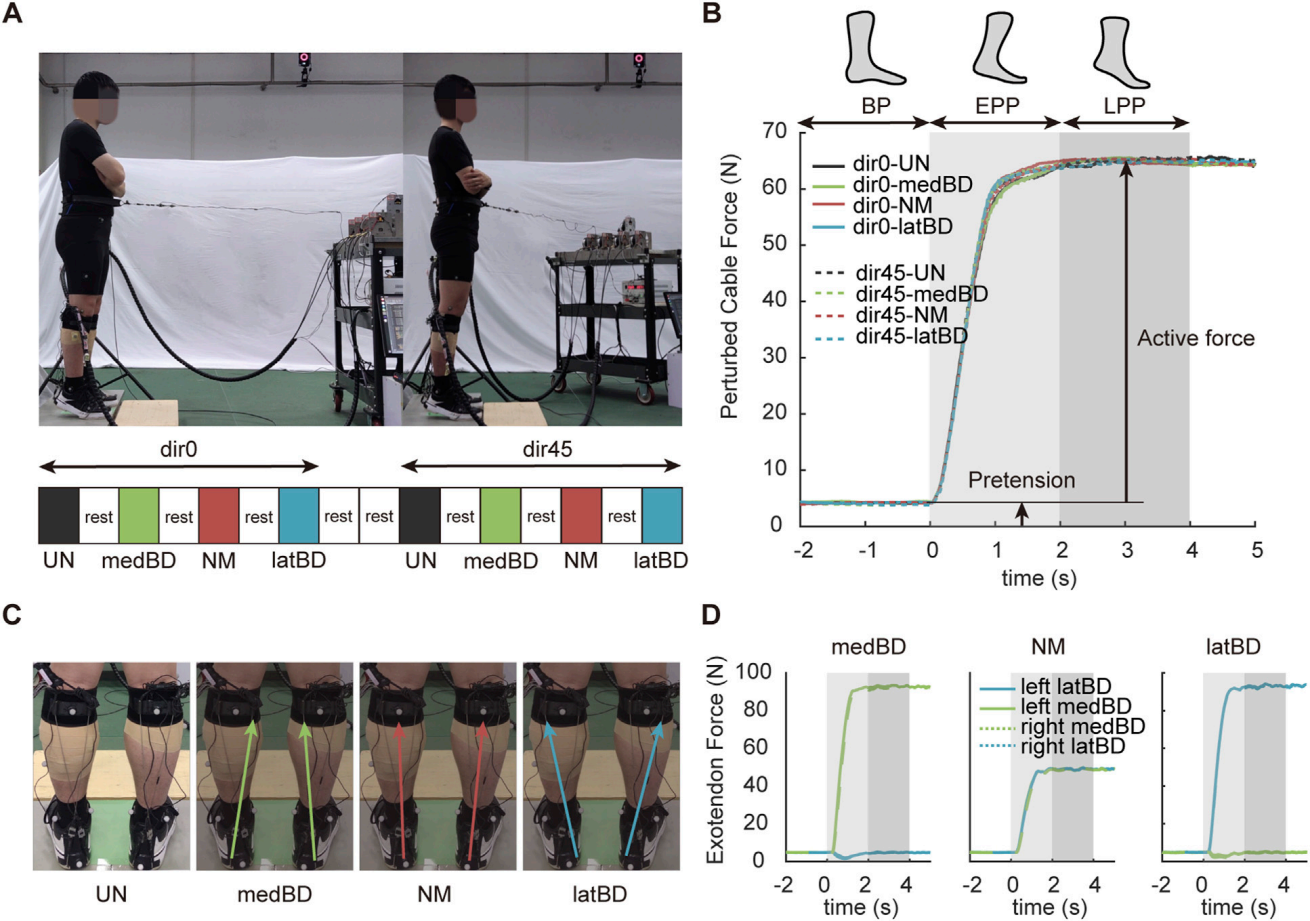
Experimental protocol. **(A)** The trial conditions of two perturbed directions and four assistive modes and their sequence. The standard forward direction is represented by dir0, while the left-forward direction is represented by dir45. The black, yellow, red, and blue represent the unpowered (UN), medial boundary (medBD), normal (NM) and lateral boundary (latBD) modes, respectively. **(B)** The measured perturbed force trajectories under eight conditions for a typical subject. The solid lines represent dir0 condition, while the dash lines represent dir45 condition. Light gray shaded region indicates the EPP period while the dark gray indicated the LPP period. **(C)** The total force vectors of four assistive modes. **(D)** The measured assistive force trajectories of three powered assistive modes for a typical subject under dir0 condition. The yellow and blue represent lateral and medial boundary cable forces, respectively. The solid lines represent the left exoskeleton, while the dash lines represent the right.

If a coordinate system was attached to the heel in the exoskeleton plane (Figure 1C), the total force vector $f_{\text{Lexo}}$ could be decomposed into medial-lateral and upward-downward components (Equation 1):

$$f_{\text{Lexo}}=f_{\text{Lexo,ML}}\cdot e_{\text{x}}+f_{\text{Lexo,UD}}\cdot e_{\text{y}}$$ (1)

where, each component was the combined effect of the forces on the two cables (Equations 2, 3):

$$f_{\text{Lexo,ML}}=\left(f_{\text{Lexo,medBD}}-f_{\text{Lexo,latBD}}\right)\cdot \sin\frac{\theta }{2}$$ (2)

$$f_{\text{Lexo,UD}}=\left(f_{\text{Lexo,medBD}}+f_{\text{Lexo,latBD}}\right)\cdot \cos\frac{\theta }{2}$$ (3)

where, ${\text{f}}_{\text{Lexo,medBD}}$ was the force scalar in the cable leaning to the medial side of the body, which originated from the medial side of the left tibia and was inserted into the lateral side of the left heel. Instead, ${\text{f}}_{\text{Lexo,latBD}}$ was the force scalar in the cable leaning to the lateral side of the body, which originated from the lateral side of the left tibia and was inserted into the medial side of the left heel. Then, the virtual acting point $p_{\text{x}}$ , direction angle $\beta_{\text{y}}$ , and magnitude ${\text{f}}_{\text{Lexo}}$ of the total force vector were as follows (Equations 4–6):

$$p_{\text{x}}=-H\cdot \frac{f_{\text{Lexo,ML}}}{f_{\text{Lexo,UD}}}$$ (4)

$$\beta_{\text{y}}=arctan\frac{f_{\text{Lexo,ML}}}{f_{\text{Lexo,UD}}}$$ (5)

$$f_{\text{Lexo}}=\sqrt{{f_{\text{Lexo,medBD}}}^{2}+{f_{\text{Lexo,latBD}}}^{2}}$$ (6)

where, H is the distance from the cable crossing point to the middle of the heel. Thus, the orientation of the total force and be adjusted easily by changing the relative magnitudes of two cable forces (Figure 2C, D). Then, the torque ratio of the exoskeleton will change as well.

### 2.2 Experimental protocol

Seven healthy subjects took part in the experiment. The experimental protocol was approved by the Huazhong University of Science and Technology Committee, and informed consent was obtained from all participants. In this study, subjects were asked to try to maintain their balance without stepping while receiving horizontal pulling perturbations on the pelvis.

We set two pulling directions (Figure 2A): one is standard forward (dir0), and another is left-forward (dir45) deviating from standard forward with an angle of 45°. Subjects using our ankle-foot exoskeleton with four assistive modes (Figure 2C): unpowered mode (UN), boundary mode biased to the medial side of the body (medBD), normal mode that current exoskeleton mostly used (NM), boundary mode biased to the lateral side of the body (latBD). Thus, each subject was tested with eight conditions (Figure 2A): dir0-UN, dir0-medBD, dir0-NM, dir0-latBD, dir45-UN, dir45-medBD, dir45-NM, and dir45-latBD in sequence. To avoid fatigue, the subject was asked to have a 5-min break after each trial and an additional 5-min break after each pulling directions The equipment to apply perturbation force and cable force was a customized cable-driven off-board emulator. The hardware and force tracking control method in ROS Noetic were described in detail in our prior study (Liu et al., 2023). The control capacity of the cable-driven exoskeleton has been verified in our another prior research (Li et al., 2024).

Before all the trials, the participants were familiarized with the perturbation and assistance to adapt to their most comfortable and repeatable balance strategy. Each trial condition consisted of 12 successive pelvis perturbations. Before each trial, the subject was instructed to stand along the same foot card and with their comfortable center of pressure to replicate the initial position and center of pressure between trials as much as possible. During the trial, subjects were instructed to “try not to step” in response to the perturbations and hold their arms across the chest, while other segmental movements were not constrained, except for a tight waist suit around the pelvis.

Once the trial started, a pretension $f_{\text{pre}}=4\text{ N}$ was applied to keep the cable tight before the perturbation which did not cause significant changes in human postural sway. To make sure that the subject had achieved standing balance with the baseline tension, the first pull came at an expected time after a “ready” signal for each trial and the following pulls came with 10 s interval one by one. The compliant perturbation profile $f_{\text{desire}}\left(t\right)$ , referring to a previous study (Robert et al., 2018), was a simple ramp reaching the target force within 1 s and held for a prescribed time of 5 s before release (Figure 2B). After the cable tension was released and the subject could lean back and wait for the next pull. We cut 6 s recorded data for each perturbation for analyses, consisting of three periods: 2 s before perturbation (BP), 2 s early perturbation period (EPP), and 2 s late perturbation period (LPP) for further analysis.

Humans can adopt various muscle activation patterns under the same experimental condition, which may increase the difficulty of analysis. To reduce the randomness of human performance under the unpowered condition, the maximum desired active force $f_{\text{active\_max}}$ of the perturbation profile $f_{\text{desire}}\left(t\right)$ was designed to make humans activate their muscles as much as possible to prevent a fall. Based on a body weight normalized perturbation force ${\hat{f}}_{\text{ind}}$ (Equation 8), which was required to initiate a step (Robert et al., 2018), the $f_{\text{active\_max}}$ for each subject was pre-tested to make sure humans can keep balance without stepping when the direction of perturbation is forward (Equation 7).

$$f_{\text{active\_max}}=\frac{{\hat{f}}_{\text{ind}}}{100}\times mg$$ (7)

$${\hat{f}}_{\text{ind}}=\frac{2434}{t_{\text{p}}}+7.69$$ (8)

where m was body mass for each subject and g was the acceleration of gravity. The relationship between ${\hat{f}}_{\text{ind}}$ and the perturbation duration $t_{\text{p}}$ was established by fitting a hyperbolic function with a positive horizontal asymptote with experimental data (Robert et al., 2018). The mass, height, and desired active force for each subject were shown in Table 1. The shape and magnitude of the perturbation profile were the same for dir0 and dir45 conditions (Figure 2B).

**TABLE 1:** The mass, height, and desired active force of the perturbation for each subject.

Subject index	Mass (kg)	Height (cm)	Desired active force (N)
1	66.3	175	62
2	72.0	170	55
3	72.0	180	66
4	62.8	172	53
5	59.4	177	47
6	58.5	178	47
7	68.0	172	58

During the pre-tests, the ankle moment showed the same profile shape as the perturbation, thus the assistive force profile applied by each Bowden cable was designed to be the same shape as the perturbation profile. The exoskeleton’s ability to withstand certain forces was validated by another pre-test. The scalar sum of two cable forces per leg was gradually increased in 10N steps starting at 10N until the strap slipping down from the shank. The maximum value was about 100N, and was then used as a constraint when designing three assistive modes. We controlled the scalar sum of two cable forces of the left exoskeleton to be 100 N for all subjects and each rope has a pretension of 5 N. The pair of desired assistive forces were different for each assistive mode were shown in Table 2. The right exoskeleton was controlled the same as the left (Figure 2D). Detection and triggering methods were employed, where the cable applying the perturbation was in series with a force sensor. Perturbation detection occurred when the cable force exceeded 10N with a time interval longer than 5 s since the stop time of the last perturbation. Upon detection, an assistive force profile was triggered.

**TABLE 2:** Assistive modes and desired active force pairs of the exoskeleton cables.

Exoskeleton assistive Mode	Left		Right	
	latBD (N)	medBD (N)	medBD (N)	latBD (N)
UN	0	0	0	0
medBD	0	90	90	0
NM	45	45	45	45
latBD	90	0	0	90

### 2.3 Measurement

There were 32 bony landmarks on the human body: 22 on the lower limbs and pelvis, 5 on the trunk, 3 along the perturbation rope, and 2 on the exoskeleton brace. All the marker trajectories were collected at 100 Hz using a video motion capture system with eight cameras (Vicon, Oxford Metrics Ltd., United Kingdom) and customized software (Nexus, Oxford Metrics Ltd., United Kingdom).

Five surface electromyographic electrodes (SX230, Biometrics Ltd., United Kingdom) were used to record surface electromyographic signals (EMG) on PERL, TA, LGAS, MGAS and SOL of the left leg. The electrodes were attached to the human body according to SENIAM guidelines (Hermens et al., 1999). Two force platforms (BMS400600, AMTI Inc., United States) were used to collect the ground reaction force and the center of pressure under each foot. These force platforms and electrodes were connected to the Vicon Nexus and the signals were captured at 1,000 Hz.

The ROS system collected five load cell data at 100 Hz, one for the perturbation force and the other for the assistive forces. To achieve synchronous data acquisition, Vicon Nexus was triggered to start and stop data capture via the UDP protocol in the ROS system. In addition, the accurate delay can be obtained by comparing the ground reaction force measured by ROS to that recorded by Vicon Nexus.

### 2.4 Data processing

**FIGURE 3:**
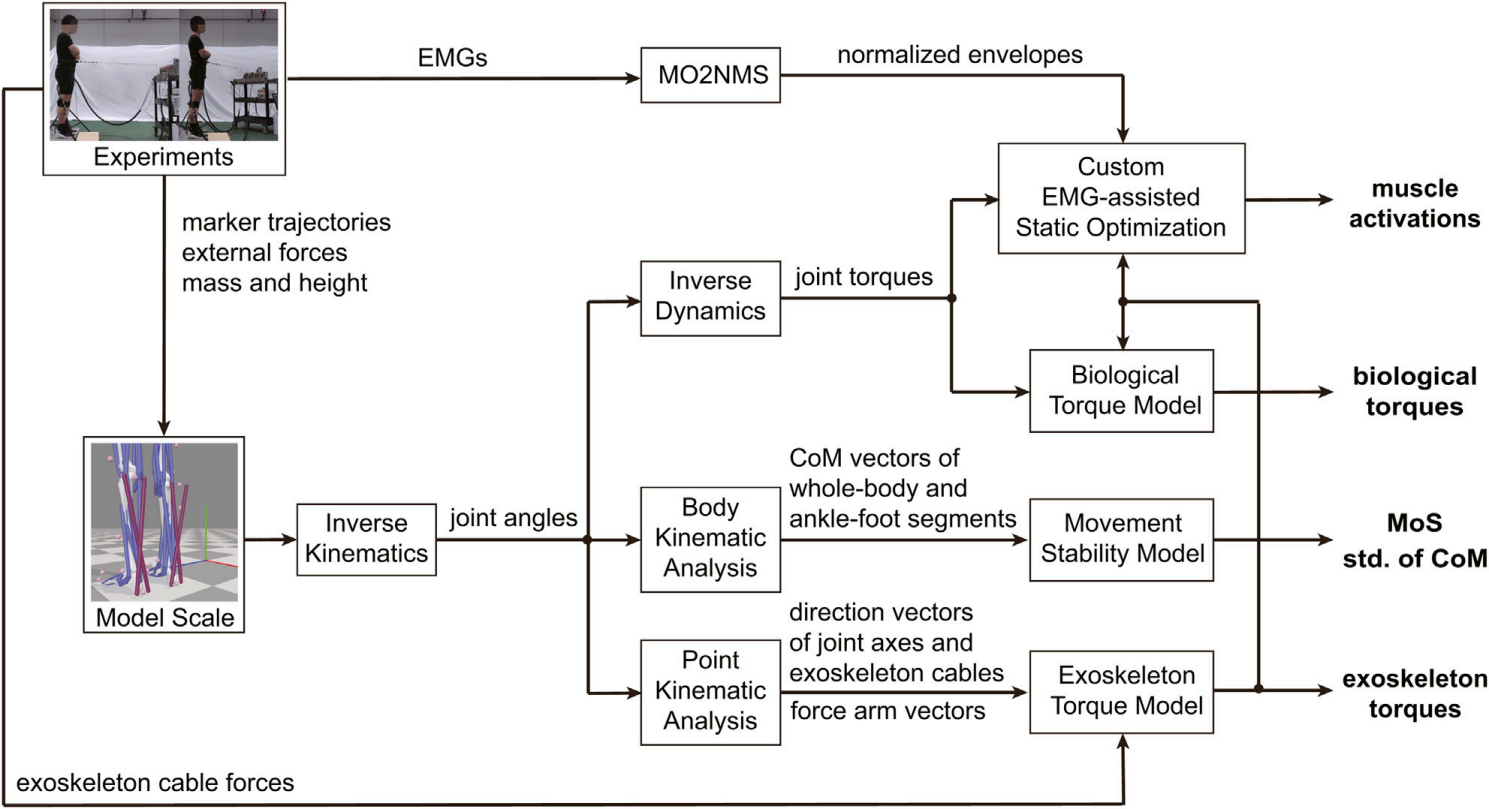
Workflow of modeling and data processing. The variables used for statistical analyses were highlighted in bold. The MoS represents the margin of stability, the CoM represents the center of mass, and the std. Represents the standard deviation. The ankle-foot segments were divided into three parts: the talus, calcaneus, and toes, according to the subtalar and metatarsophalangeal joints.

Before conducting data analysis, we calculated the root mean squared error (RMSE) of the measured perturbation and assistive forces relative to the desired profile. This step was taken to ensure that force tracking errors have minimal impact on human performance. The left leg was used as a representative in this study because one of the directions of perturbation was left-forward. We used OpenSim 4.4 (Delp et al., 2007) and a musculoskeletal model described by Scott et al. (Uhlrich et al., 2022). The model had 23 degrees of freedom and 40 musculotendon actuators on the lower extremity. The talocrural and subtalar joints each have one degree of rotational freedom. The generic model was scaled using the OpenSim Scale tool to match bony landmark measurements from a 5 s static trial, and the virtual markers on the model were moved to match experimental marker locations from this trial (Figure 3). To ensure the following process accuracy, the overall RMS marker error should be less than 1 cm, with maximum marker errors typically less than 2 cm after scaling. Additionally, the static pose should be typical, with an ankle angle of less than 5 ° and a hip flexion angle of less than 10 ° . The kinematics were then estimated using the Inverse Kinematics tool to keep the error between measured and virtual marker positions to 4 cm for the maximum and 2 cm for the RMS.

Joint torques were calculated using the Inverse Dynamics tool in OpenSim, with low-pass-filtered kinematics (6 Hz, $6^{\text{th}}$ order, zero-phase shift Butterworth) and ground reaction forces, as well as perturbation forces (6 Hz, $4^{\text{th}}$ order, zero-phase shift Butterworth) as input. Using Point Kinematic Analysis tool in OpenSim, the kinematics of the points that we concerned can be obtained. The direction vectors of left talocrural axis $e_{\text{Ltal}}$ , left subtalar axis $e_{\text{Lsub}}$ and the exoskeleton cables $e_{\text{Lexo,medBD}}$ , $e_{\text{Lexo,latBD}}$ can be obtained easily using the coordinate trajectories of those points. Additionally, the force arm vectors from the left talocrural and subtalar rotation centers to the exoskeleton’s insertion points on the heels, including $r_{\text{LtalO}2\text{Lexo,latBD}}$ , $r_{\text{LtalO}2\text{Lexo,medBD}}$ , $r_{\text{LsubO}2\text{Lexo,latBD}}$ , and $r_{\text{LsubO}2\text{Lexo,medBD}}$ can be calculated respectively.

The exoskeleton’s talocrural torque was calculated by multiplying two cable force vectors with corresponding force arm vectors (Equations 9, 10), and then projecting the sum of those on the joint axis (Equation 11):

$$\tau_{\text{Lexo,latBD,Ltal}}=r_{\text{LtalO}2\text{Lexo,latBD}}\times \left(f_{\text{Lexo,latBD}}\cdot e_{\text{Lexo,latBD}}\right)$$ (9)

$$\tau_{\text{Lexo,medBD,Ltal}}=r_{\text{LtalO}2\text{Lexo,medBD}}\times \left(f_{\text{Lexo,medBD}}\cdot e_{\text{Lexo,medBD}}\right)$$ (10)

$$\tau_{\text{Lexo,Ltal}}=\left(\tau_{\text{Lexo,latBD,Ltal}}+\tau_{\text{Lexo,medBD,Ltal}}\right)\cdot e_{\text{Ltal}}$$ (11)

It was the same with the subtalar joint (Equations 12–14):

$$\tau_{\text{Lexo,latBD,Lsub}}=r_{\text{LsubO}2\text{Lexo,latBD}}\times \left(f_{\text{Lexo,latBD}}\cdot e_{\text{Lexo,latBD}}\right)$$ (12)

$$\tau_{\text{Lexo,medBD,Lsub}}=r_{\text{LsubO}2\text{Lexo,medBD}}\times \left(f_{\text{Lexo,medBD}}\cdot e_{\text{Lexo,medBD}}\right)$$ (13)

$$\tau_{\text{Lexo,Lsub}}=\left(\tau_{\text{Lexo,latBD,Lsub}}+\tau_{\text{Lexo,medBD,Lsub}}\right)\cdot e_{\text{Lsub}}$$ (14)

Then, the exoskeleton torque ratio can be obtained (Equation 15):

$$\lambda_{\text{Lexo}}=\frac{\tau_{\text{Lexo,Lsub}}}{\tau_{\text{Lexo,Ltal}}}$$ (15)

The biological torques (Equations 16, 17) and corresponding torque ratio (Equation 18) were estimated by subtracting the exoskeleton torques from the resultant torques calculated from the Inverse Dynamics (Collins et al., 2015):

$$\tau_{\text{Lbio,Ltal}}={\tau_{\text{Ltal}}-\tau }_{\text{Lexo,Ltal}}$$ (16)

$$\tau_{\text{Lbio,Lsub}}=\tau_{\text{Lsub}}-\tau_{\text{Lexo,Lsub}}$$ (17)

$$\lambda_{\text{Lbio}}=\frac{\tau_{\text{Lbio,Lsub}}}{\tau_{\text{Lbio,Ltal}}}$$ (18)

The recorded EMG signals were processed by MOtoNMS v2.2 (Mantoan et al., 2015) to get the normalized envelope of the EMG. The raw data were first band-pass-filtered (30–300 Hz, $2^{\text{nd}}$ order) to retain the EMG signal content. Then they were rectified by taking the absolute value and low-pass-filtered (6 Hz, $2^{\text{nd}}$ order) to create an envelope. To conduct between-subject analysis, the activation of each muscle was normalized to the maximum activation among the corresponding EMG envelopes from all trials of each subject.

We implemented a custom EMG-assisted Static Optimization provided by Scott et al. (Uhlrich et al., 2022) in MATLAB (R2022a, MathWorks, United States) for estimating activations of the unmeasured muscles, such as TP. We modified the optimization by adding exoskeleton assistance in torque tracking and adding constraints to ensure control of the measured muscles tracking the normalized EMG. The right leg muscles were ignored to complete the simulation quickly. The optimization problem was formulated as Equations 19–23:

$$\begin{array}{ll} \underset{c_{\text{Lm},i},\;c_{\text{Lr},j1},\;c_{\text{Rr},j2},\;c_{\text{Tr},j3}}{\min} & \overset{40}{\underset{i=1}{\sum }}\left(w_{\text{Lm},i}\cdot {c_{\text{Lm},i}}^{2}\right)\\ & \;+\overset{7}{\underset{j1=1}{\sum }}\left(w_{\text{Lr},j1}\cdot {c_{\text{Lr},j1}}^{2}\right)+\overset{7}{\underset{j2=1}{\sum }}\left(w_{\text{Rr},j2}\cdot {c_{\text{Rr},j2}}^{2}\right)\\ & \;+\overset{9}{\underset{j3=1}{\sum }}\left(w_{\text{Tr},j3}\cdot {c_{\text{Tr},j3}}^{2}\right) \end{array}$$ (19)

subject to:

$$\left(\tau_{\text{m}}+\tau_{\text{r}}+\tau_{\text{exo}}\right)-\tau_{\text{ID}}=0$$ (20)

$$c_{k}=\left|c_{\text{Lm},k}-a_{\text{Lm},k}\right|<0.01\;\left(k=1,2,3,4,5\right)$$ (21)

$${0\leq c}_{\text{Lm},i}\leq 1$$ (22)

$$0\leq c_{\text{Lr},j1},0\leq c_{\text{Rr},j2},0\leq c_{\text{Tr},j3}$$ (23)

where $c_{\text{Lm},i},c_{\text{Lr},j1},c_{\text{Rr},j2},c_{\text{Tr},j3}$ were the controls of actuators. L, R, and T indicated the left leg, right leg, and trunk. The muscle, reserve, and exoskeleton actuators were represented by m, r and exo. The index of the muscle on the left leg was i , while j1 , j2 , and j3 were the index of the reserve actuator on the left leg, right leg, and trunk, respectively. The weight $w_{\text{Lm},i}$ , $w_{\text{Lr},j1}$ were set as 1 and $w_{\text{Rr},j2}$ , $w_{\text{Tr},j3}$ were set as 100 based on experience. The size of the torque vector τ was 23×1. The normalized EMG envelopes were represented by $a_{\text{Lm},k}$ where k was the index of five muscles.

To evaluate how the exoskeleton enhance human resistance to perturbation in forward direction within fan-shaped region of pelvis horizontal plane, the margin of stability (MoS) (Hof et al., 2005), the minimum distance from the extrapolated centre of mass (XcoM) to the boundaries of the base of support (BoS), was modified to two dimension in horizontal plane and used as a measure of body stability (Equations 24, 25):

$$MoS=\frac{\left\|\left(p_{\text{bos,L,AL}}-p_{\text{bos,L,AM}}\right)\times p_{\text{xcom}}\right\|}{\left\|p_{\text{bos,L,AL}}-p_{\text{bos,L,AM}}\right\|}$$ (24)

$$p_{\text{xcom}}=p_{\text{com}}+\frac{{\dot{p}}_{\text{com}}}{\sqrt{g/l}}$$ (25)

where p was the vector in the horizontal plane. The anterior BoS was defined by the connecting line of the anterior medial (AM) and anterior lateral (AL) points of the BoS. The g was the gravity acceleration, and l represented the distance between the center of mass and the posterior medial (PM) point of BoS projected into the saggital plane. In addition, the standard deviation of the center of pressure (CoP) was also used as a body stability metric.

To evaluate foot-ankle stability, the ankle-foot segments were divided into three parts: the talus, calcaneus, and toes, according to the subtalar and metatarsophalangeal joints. The standard deviation of center of mass of each segmental part was used as the metric for preliminary analysis. The kinematics of the points of BoS can be obtained using the Point Kinematic Analysis tool, while the kinematics of the center of mass of the whole body and ankle-foot segments can be obtained using the Body Kinematic Analysis tool (Figure 3).

### 2.5 Statistical analyses

We extracted the average values during the LPP to characterize each measured variable, including exoskeleton torques, biological torques, and the corresponding torque ratio. The normalized EMG and optimized muscle activations were also analyzed. The two-sided Jarque-Bera test was used to confirm the normal distribution for various metrics. The two-sided paired t -test was adopted to make pairwise comparisons among four assistive modes and among two perturbation directions. First, we compared the biological torques, ratio, and muscle activations under the dir0-UN condition with the dir45-UN to explore the effect of the perturbation direction on the human natural ankle-foot strategy. Then, the exoskeleton torques and ratio with medBD and latBD assistive modes were compared to those with NM respectively, in order to validate the torque-ratio adjustable range of the exoskeleton. Lastly, the MoS, the standard deviation of CoP and foot segmental CoM, the biological torques, ratio, and muscle activations with the UN, medBD, NM, and latBD assistive modes were compared with each other to find out the changes in body stability, human ankle-foot strategy with exoskeleton assistance, and the differences between the adjustable exoskeleton and the traditional exoskeleton. Besides, two one-way repeated ANOVA tests were conducted on the perturbed force among 4 conditions of dir0 and dir45, respectively. The Bartlett tests were conducted to ensure multiple samples with equal variances. All statistical analyses were performed in the software MATLAB (R2022a, MathWorks, United States), and the significance levels for all analyses were set at α= 0.05.

## 3 Results

### 3.1 Force tracking performance

The mean values of RMSE between desired and measured forces during LPP are shown in Table 3. The RMSE values of all perturbed forces were less than 3 % of the average perturbed force 59N. Among the 4 conditions of dir0, there was no significant difference for perturbed forces (one-way repeated ANOVA, p> 0.1), and so were the 4 conditions of dir45 (one-way repeated ANOVA, p> 0.05). Compared to the same assistive mode between dir0 and dir45, the differences were also not significant (two-sided paired t -test, p> 0.1). There were three desired assistive forces: 5N, 50N, and 95N, which were the sum of pre-tension and the desired active force in Table 2. , and the distance of three desired assistive forces was 45N. The RMSE values relative to 45N was less than 7 % . Thus, the perturbed force tracking error has little influence on human response, and the assistive force tracking error has little effect on the different human responses to the three assistive modes.

**TABLE 3:** The mean and standard deviation of the root-mean-squared-error (RMSE) values between measured and desired forces during LPP, including perturbed forces and four assistive forces, The percentage represents the mean value relative to the desired perturbed force (59N) and the distance (45N) between the three desired assistive forces, respectively.

	The RMSE of perturbed force (N)	The RMSE of assistive force (N)			
Condition	Pelvis	Left		Right	
		latBD	medBD	medBD	latBD
dir0-UN	1.7 ± 0.2(3 % )	NaN	NaN	NaN	NaN
dir0-medBD	1.4 ± 0.3(2 % )	0.5 ± 0.1(1 % )	2.5 ± 0.1(6 % )	2.6 ± 0.2(6 % )	0.6 ± 0.2(1 % )
dir0-NM	1.3 ± 0.4(2 % )	1.5 ± 0.3(3 % )	1.5 ± 0.2(3 % )	1.5 ± 0.2(3 % )	1.8 ± 0.3(4 % )
dir0-latBD	1.3 ± 0.4(2 % )	2.5 ± 0.2(6 % )	0.5 ± 0.2(1 % )	0.5 ± 0.2(1 % )	2.9 ± 0.5(7 % )
dir45-UN	1.5 ± 0.4(3 % )	NaN	NaN	NaN	NaN
dir45-medBD	1.3 ± 0.2(2 % )	0.5 ± 0.1(1 % )	2.7 ± 0.2(6 % )	2.6 ± 0.3(6 % )	0.5 ± 0.1(1 % )
dir45-NM	1.1 ± 0.2(2 % )	1.5 ± 0.1(3 % )	1.6 ± 0.2(4 % )	1.4 ± 0.2(3 % )	1.8 ± 0.2(4 % )
dir45-latBD	1.3 ± 0.2(2 % )	2.7 ± 0.3(6 % )	0.5 ± 0.2(1 % )	0.4 ± 0.1(1 % )	3.1 ± 0.6(7 % )

### 3.2 Biological torques and muscle activations without assistance

**FIGURE 4:**
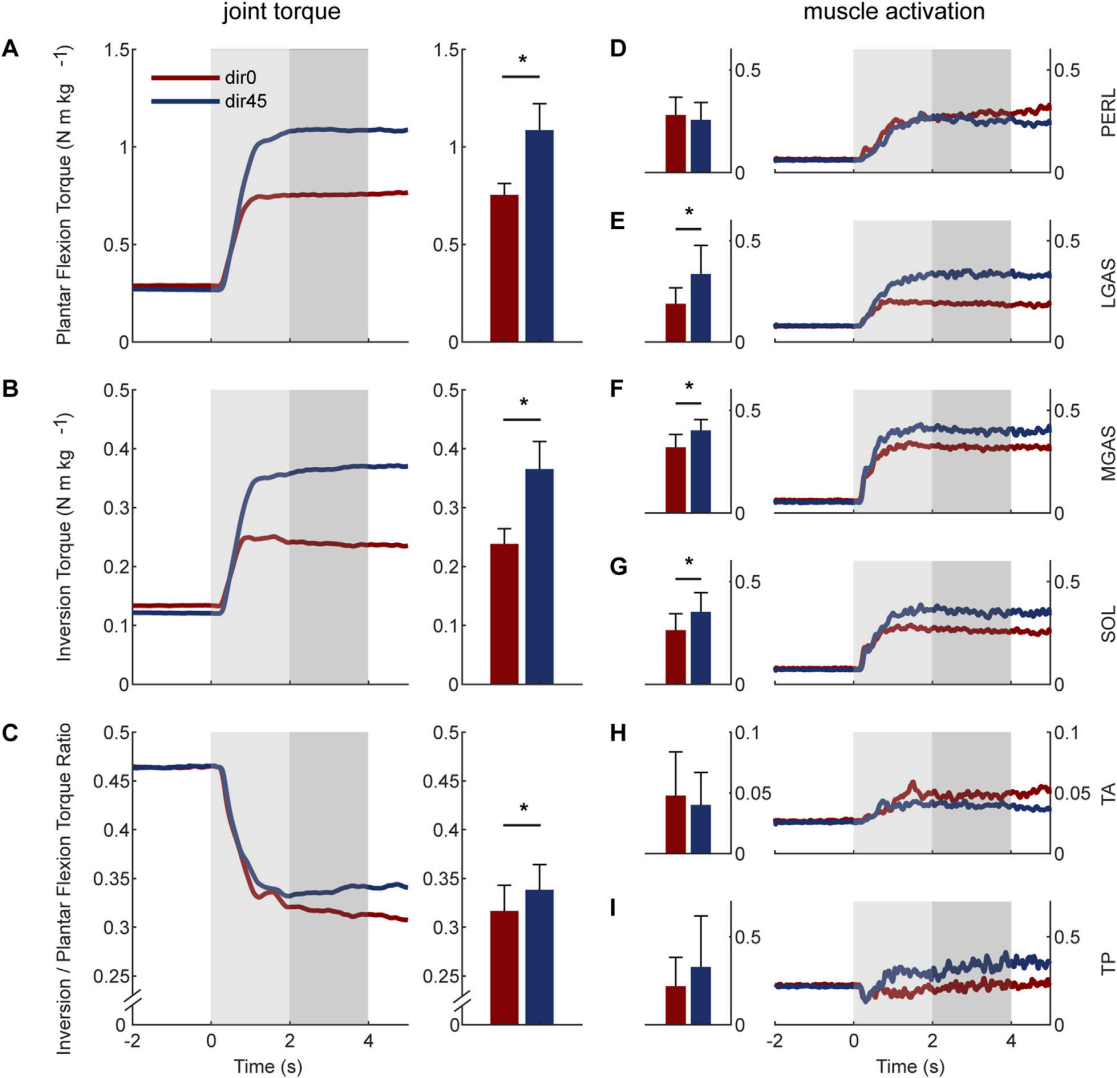
The natural torques and muscle activations of the human ankle-foot under dir0 and dir45 perturbations on the pelvis: **(A)** plantar flexion torque; **(B)** inversion torque; **(C)** the torque ratio of inversion to plantar flexion; **(D)** PERL; **(E)** LGAS; **(F)** MGAS; **(G)** SOL; **(H)** tibialis anterior (TA); **(I)** TP. The average values during the LPP period are shown in the middle two columns. The error bars indicate the standard deviation. Asterisks indicate statistically significant differences ( n= 7, two-sided paired t -test, p< 0.05), except for the SOL ( n= 6, two-sided paired t -test, p< 0.05).

The human natural ankle-foot strategy at joint level is shown in the left two columns of Figure 4. When resisting dir45 perturbation, subjects increased the average ankle-foot torque during LPP compared with dir0. The increase for plantar flexion torque was 44.9 % (0.33 N ⋅ m ⋅ k ${\text{g}}^{-1}$ ; n= 7, two-sided paired t -test, p< 0.001), while inversion torque increased 53.4 % (0.13 N ⋅ m ⋅ k ${\text{g}}^{-1}$ ; n= 7, two-sided paired t -test, p< 0.001). The torque ratio of inversion to plantar flexion was increased from 0.32 under dir0 to 0.34 under dir45 (6.9 % ; n= 7, two-sided paired t -test, p= 0.016).

The human natural ankle-foot strategy at muscle level is shown in the right two columns of Figure 4. Compared with dir0, the LGAS and MGAS activations under dir45 were increased by 77.9 % and 26.1 % ( n= 7, two-sided paired t -test, p< 0.01), respectively. There was a trend toward an increase in the SOL muscle activation compared with the dir0 by 34.4 % ( n= 6, two-sided paired t -test, p= 0.03). It was found no significant difference in PERL, TA, and TP between the two conditions ( n= 7, two-sided paired t -test, p> 0.1).

### 3.3 Exoskeleton-assisted torques

**FIGURE 5:**
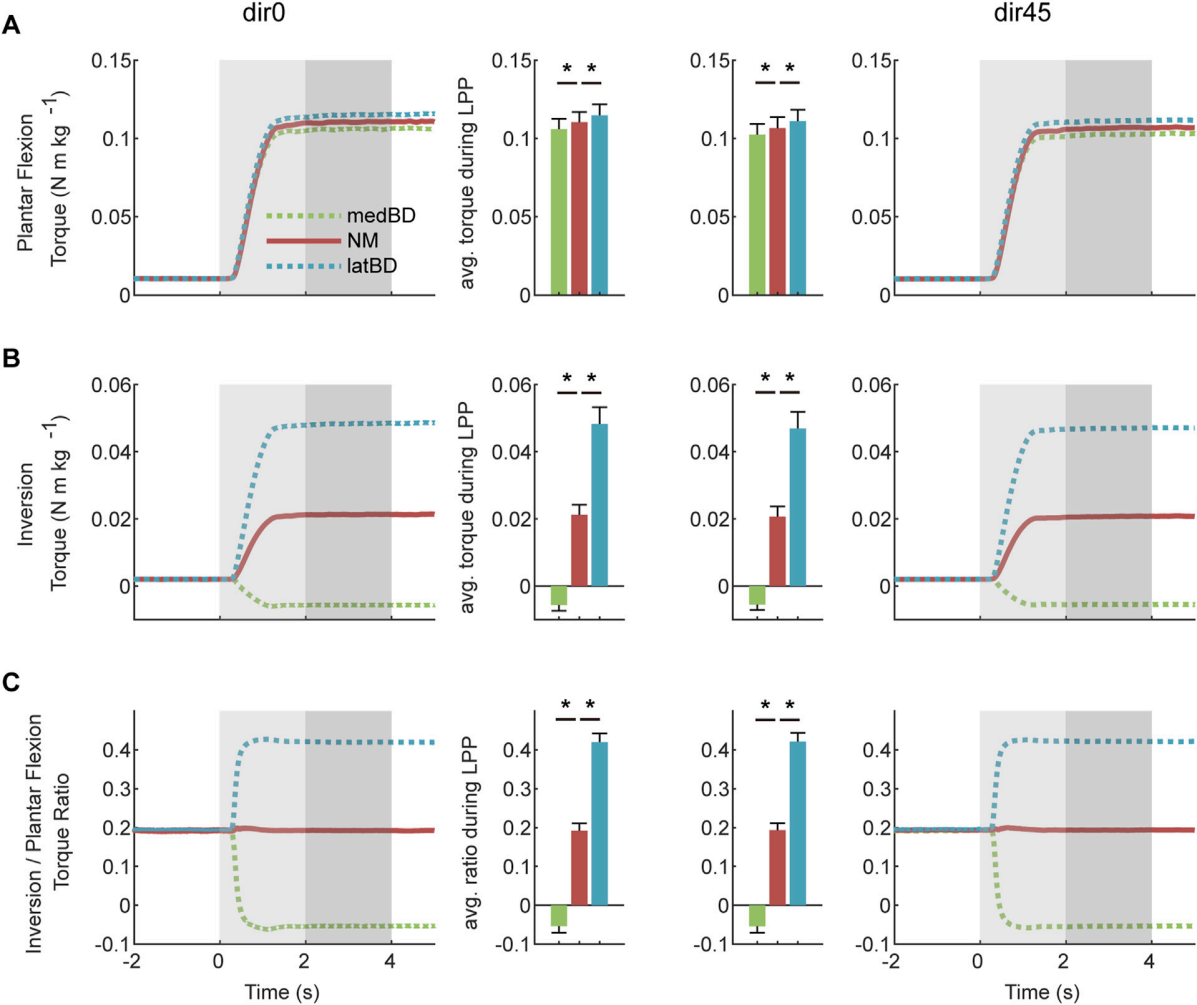
The exoskeleton-assisted torques under dir0 and dir45 conditions: **(A)** plantar flexion torque; **(B)** inversion torque; **(C)** torque ratio of inversion to plantar flexion. The left two columns indicate dir0 condition, while the right two indicate dir45. The red, green, and blue represent NM, medBD, and latBD, respectively. The error bars indicate the standard deviation. Asterisks indicate statistically significant differences ( n= 7, two-sided paired t -test, p< 0.05).

The assistive torques generated by NM, medBD and latBD modes of the exoskeleton are shown in Figure 5. Compared with NM mode, the plantar flexion torque decreased by 4.1 % for medBD and increased by 4.0 % for the latBD ( n= 7, two-sided paired t -test, p< 0.001) under dir0 perturbation. For inversion toque, there was a 126.6 % reduction for the medBD and a 126.9 % increase for the latBD ( n= 7, two-sided paired t -test, p< 0.001) compared with NM mode. Thus, the torque ratio reduced by 128.0 % for the medBD and increased by 118.3 % for the latBD ( n= 7, two-sided paired t -test, p< 0.001). When resisting dir45 perturbation, the significant difference between NM and BD modes persisted, and the relative magnitudes were almost the same.

The NM mode could offer about 0.110 N ⋅ m ⋅ k ${\text{g}}^{-1}$ plantar flexion torque, 0.021 N ⋅ m ⋅ k ${\text{g}}^{-1}$ inversion torque and 0.192 torque ratio of inversion to plantar flexion for each leg under dir0. Compared with dir0, the plantar flexion torque under dir45 reduced significantly (−3.5 % ; n= 7, two-sided paired t -test, p= 0.001), while the inversion showed a reduction trend ( n= 7, two-sided paired t -test, p= 0.07). There was no significant difference of torque ratio between two perturbation conditions.

The assistive modes between the medBD and latBD could offer plantar flexion torque ranging from 0.106 to 0.115 N ⋅ m ⋅ k ${\text{g}}^{-1}$ , inversion torque ranging from −0.006–0.048 N ⋅ m ⋅ k ${\text{g}}^{-1}$ , and the torque ratio ranging from −0.054 to 0.420 under dir0. Compared with dir0, the plantar flexion torque under dir45 reduced significantly (−3.3 % , −3.2 % ; n= 7, two-sided paired t -test, p< 0.001, p= 0.001) for the medBD and latBD modes, respectively. For inversion torque, there was no significant difference between two perturbation conditions for the medBD mode ( n= 7, two-sided paired t -test, p= 0.17), while there was a significant decrease for the latBD mode (−2.8 % ; n= 7, two-sided paired t -test, p< 0.01). Together, the torque ratio showed no significant difference for the medBD ( n= 7, two-sided paired t -test, p> 0.1), while there was an increase trend for the latBD ( n= 7, two-sided paired t -test, p= 0.059).

### 3.4 Changes of body stability with assistance

The average value of MoS is shown in Figure 9A. Compared to the UN mode, the powered assistive modes significantly increased MoS by 6.9 % , 6.6 % , and 5.9 % for medBD, NM, and latBD, respectively ( n= 7, two-sided paired t -test, p< 0.05) under dir0 perturbation. The increases were 5.3 % , 5.9 % , and 6.5 % when resisting dir45 perturbation ( n= 7, two-sided paired t -test, p< 0.1). Among powered modes, there was no significant difference between NM and BD modes ( n= 7, two-sided paired t -test, p> 0.1).

For the standard deviation of COP, Figure 9B shows the component in the anterior/posterior direction, and Figure 9C shows the medial/lateral component. Compared to the UN mode, the powered assistive modes significantly decreased the standard deviation of COP by about 40 % in A/P and 64 % in M/L ( n= 7, two-sided paired t -test, p< 0.05) under dir0 perturbation. However, when resisting dir45 perturbation, only NM caused a significant decrease of about 24 % in A/P ( n= 7, two-sided paired t -test, p< 0.05) and 38 % in M/L ( n= 7, two-sided paired t -test, p< 0.1). Among powered modes, there was no significant difference between NM and BD modes for the standard deviation of COP ( n= 7, two-sided paired t -test, p> 0.1), except for the A/P component’s difference between medBD and NM under dir45 perturbation ( n= 7, two-sided paired t -test, p< 0.1).

The standard deviation of the COM of talus, calcaneus, and toes segments are shown in Figure 10A–C, respectively. Compared to the UN mode, the powered assistive modes significantly decreased the standard deviation by 42 % to 64 % ( n= 7, two-sided paired t -test, p< 0.1) under dir0 perturbation. When resisting dir45 perturbation, the decreases ranged from about 31 % to 54 % and were not significant for some standard deviations of BD modes ( n= 7, two-sided paired t -test, p> 0.1). Among powered modes, there was no significant difference between NM and BD modes ( n= 7, two-sided paired t -test, p> 0.1).

### 3.5 Biological torques and muscle activations across assistive modes

#### 3.5.1 Biological torques

**FIGURE 6:**
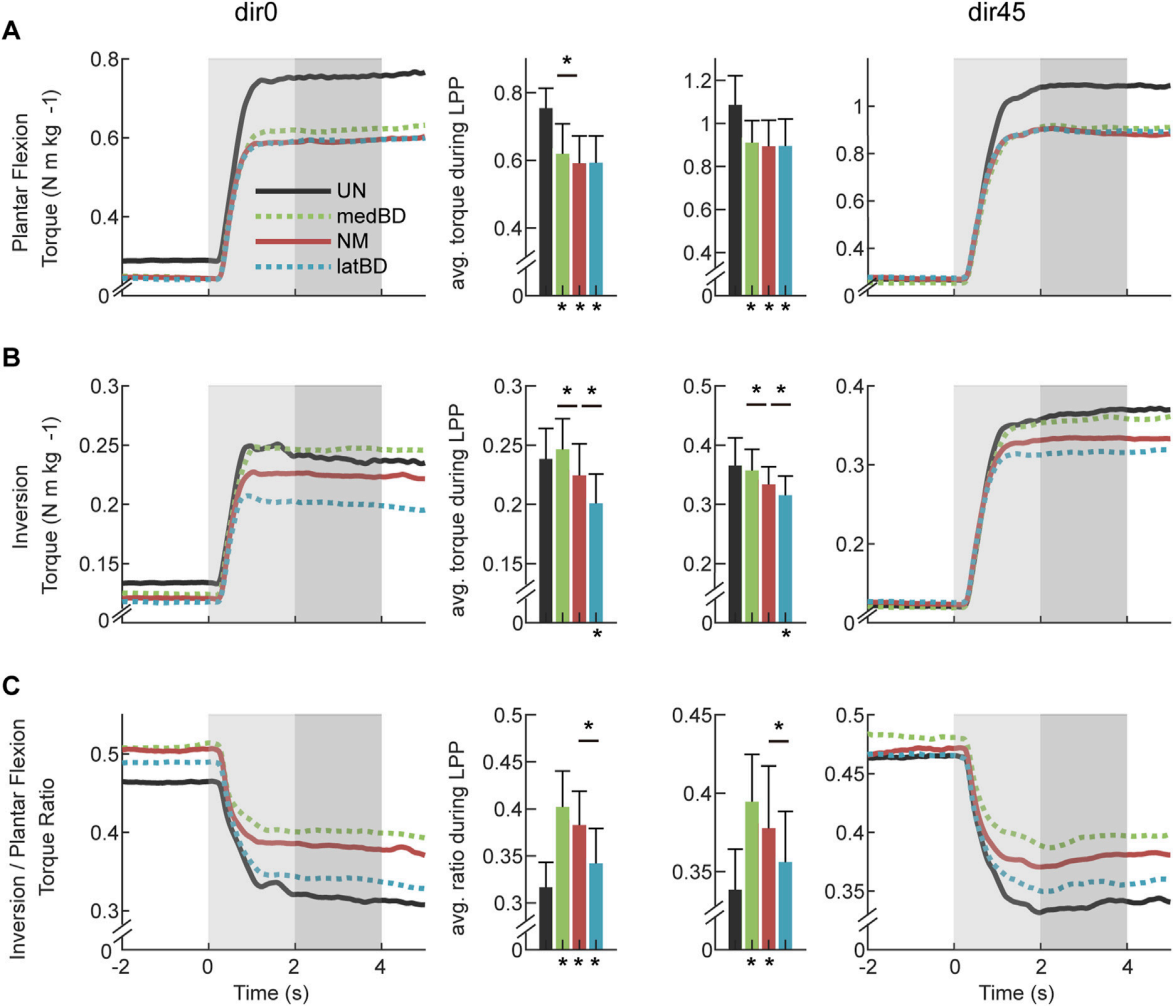
The biological torques with assistance under dir0 and dir45 conditions: **(A)** plantar flexion torque; **(B)** inversion torque; **(C)** torque ratio of inversion to plantar flexion. The black, red, green, and blue represent UN, NM, medBD, and latBD, respectively. The average values during the LPP period are shown in the bars. The error bars indicate the standard deviation. Asterisks below the bars indicate a statistically significant difference compared with UN mode ( n= 7, two-sided paired t -test, p< 0.05). Asterisks above the bars represent the significant difference compared with NM mode ( n= 7, two-sided paired t -test, p< 0.05).

The biological torques assisted with exoskeleton of UN, medBD, NM, and latBD modes are shown in Figure 6. Compared with UN mode, the powered assistive modes decreased the biological plantar flexion torque by 17.9 % , 21.6 % and 21.4 % for medBD, NM and latBD, respectively ( n= 7, two-sided paired t -test, p< 0.001) under dir0 perturbation. The decrease was 16.1 % , 17.7 % and 17.6 % when resisting dir45 perturbation ( n= 7, two-sided paired t -test, p< 0.01). Among powered modes, there was no significant difference between NM and BD modes ( n= 7, two-sided paired t -test, p> 0.1), expect for a little increase from the NM to the medBD under dir0 condition (4.7 % ; n= 7, two-sided paired t -test, p< 0.05).

For the biological inversion torque, the changes from UN to powered assistive modes was 3.4 % , −5.9 % and −15.8 % for medBD, NM and latBD, respectively ( n= 7, two-sided paired t -test, p= 0.099, p= 0.087, p< 0.001) under dir0 perturbation. The changes were −2.3 % , −8.8 % and −13.7 % when resisting dir45 perturbation ( n= 7, two-sided paired t -test, p> 0.1, p= 0.056, p< 0.01). Among powered modes, the medBD was significantly larger than NM (dir0: 9.8 % , dir45: 7.1 % ; n= 7, two-sided paired t -test, p< 0.01), while the latBD was significantly smaller than NM (dir0: -10.5 % , dir45: -5.4 % ; n= 7, two-sided paired t -test, p< 0.01).

There was significant increase in the torque ratio of powered assistive modes compared with UN mode (dir0: medBD 27.0 % , NM 21.0 % , latBD 8.0 % ; dir45: medBD 16.6 % , NM 11.6 % ; n= 7, two-sided paired t -test, p< 0.05), except for the latBD under dir45 (5.2 % ; n= 7, two-sided paired t -test, p> 0.1). The torque ratio for medBD, NM and latBD were 0.40, 0.38 and 0.34 under dir0, and 0.39, 0.38 and 0.36 under dir45. The latBD was significant smaller than NM ( n= 7, two-sided paired t -test, p< 0.05), while the medBD was slightly larger than NM ( n= 7, two-sided paired t -test, p> 0.05).

#### 3.5.2 Muscle activations

**FIGURE 7:**
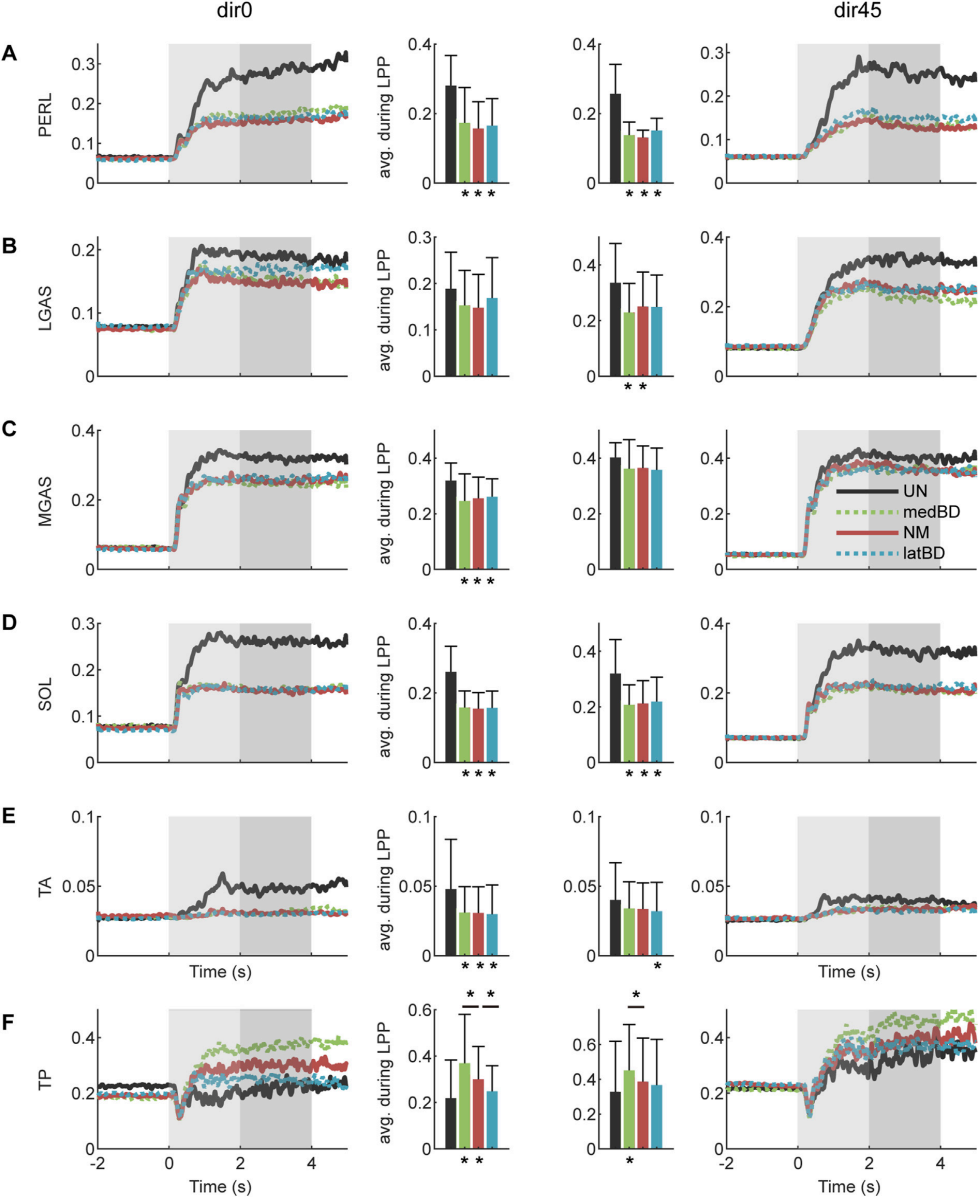
The muslce activations with assistance under dir0 and dir45 conditions: **(A)** PERL; **(B)** LGAS; **(C)** MGAS; **(D)** SOL; **(E)** TA; **(F)** TP. The black, red, green and blue represent the UN, NM, medBD, and latBD modes, respectively. The average values during LPP period are shown in the bars. The error bars indicate the standard deviation. Asterisks below the bars indicate a statistically significant difference compared with UN mode ( n= 7, two-sided paired t -test, p< 0.05). Asterisks above the bars represent the significant difference compared with NM mode ( n= 7, two-sided paired t -test, p< 0.05).

The normalized EMG envelops and optimized TP activation are shown in Figure 7. Compared with the UN, the normalized EMG envelops were significant reduced with the medBD, NM and latBD assistance ( n= 7, two-sided paired t -test, p< 0.05), except for the LGAS under dir0, the MGAS under dir45 and the TA under dir45 ( n= 7, two-sided paired t -test, p> 0.1). Among powered modes, there was no significant difference between NM and BD modes ( n= 7, two-sided paired t -test, p> 0.1).

Compared with the UN, the optimized TP activation remained no significant change for the latBD ( n= 7, two-sided paired t -test, p> 0.1), but was significant increased with the medBD and NM ( n= 7, two-sided paired t -test, p< 0.01) under dir0 condition. Compared with dir0, the TP activation under dir45 remained no significant change for the NM and latBD ( n= 7, two-sided paired t -test, p> 0.1), but was significant increased with the medBD ( n= 7, two-sided paired t -test, p< 0.05). Among powered modes, the latBD was smaller than the NM ( n= 7, two-sided paired t -test, dir0: p< 0.05, dir45: p> 0.1), while the medBD was significantly larger than the NM ( n= 7, two-sided paired t -test, dir0: p< 0.05, dir45: p< 0.01).

#### 3.5.3 Influencing factors of biological torque reduction

**FIGURE 8:**
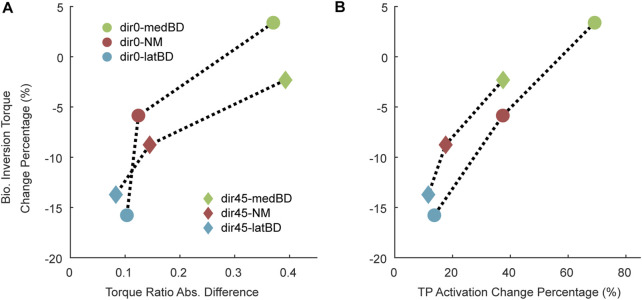
The factors influencing the biological inversion torque reduction compared with the UN mode: **(A)** the absolute differences between the biological torque ratio with the UN mode and the exoskeleton torque ratio with the powered modes; **(B)** the increased percentage of TP activation relative to the UN mode. The circle point represents the dir0 condition, while the diamond represents the dir45. The red, green and blue represent the NM, medBD, and latBD modes, respectively.

The absolute differences between the biological torque ratio under the UN mode and the exoskeleton torque ratio under powered modes are shown in Figure 8A. Meanwhile, the increase percentage of TP activation relative to the UN mode is shown in Figure 8B. The latBD mode showed the minimum torque ratio absolute difference, the minimum increase percentage of TP activation, and the maximum biological inversion torque reduction among the three powered assistive modes. On the contrary, the medBD mode showed the maximum torque ratio absolute difference, the maximum increase percentage of TP activation, and the minimum biological inversion torque reduction among the three powered modes.

## 4 Discussion

To figure out how to assist humans in resisting perturbation, it is useful to find out the human natural ankle-foot strategy. Our result showed that the human torque ratio was significantly increased from 0.32 under forward pelvis perturbation to 0.34 under left-forward perturbation. At the joint level, the increase in human inversion torque was larger than that in human plantar flexion torque, which accounted for the changes in ratio. The increased left ankle-foot torque was caused by the increased gravity bearing on the left leg. At the muscle level, the SOL, LGAS, and MGAS, which are main plantar flexors but also can provide little inversion torque, were activated more when the direction of perturbation changed to the left forward, while the main evertor PERL with little plantar flexors showed no significant changes. These changes in muscle activation patterns resulted in a larger percentage increase in human inversion torque than plantar flexion torque. The result suggests that the exoskeleton may need to have the ability to adjust its torque ratio according to the direction of perturbation for better assistance.

### 4.1 Effect of the perturbation direction on human natural ankle-foot strategy

To figure out how to assist humans in resisting perturbation, it is useful to find out the human natural ankle-foot strategy. Our result showed that the human torque ratio was significantly increased from 0.32 under forward pelvis perturbation to 0.34 under left-forward perturbation (Figure 4C). At the joint level, the increase in human inversion torque was larger than that in human plantar flexion torque, which accounted for the changes in ratio. The increased left ankle-foot torque was caused by the increased gravity bearing on the left leg. At the muscle level, the SOL, LGAS, and MGAS, which are main plantar flexors but also can provide little inversion torque, were activated more when the direction of perturbation changed to the left forward, while the main evertor PERL with little plantar flexors showed no significant changes. These changes in muscle activation patterns resulted in a larger percentage increase in human inversion torque than plantar flexion torque. The result suggests that the exoskeleton may need to have the ability to adjust its torque ratio according to the direction of perturbation for better assistance.

### 4.2 Torque-ratio-adjustable range of the exoskeleton

This study set two boundary assistive modes to validate the torque direction adjustment ability. There was a significant difference in the plantar flexion torque and the inversion torque, as well as the torque ratio between the two boundary modes. The range of plantar flexion torque was 0.106–0.115 N ⋅ m ⋅ k ${\text{g}}^{-1}$ (Figure 5A), and the inversion torque was −0.006–0.048 N ⋅ m ⋅ k ${\text{g}}^{-1}$ (Figure 5B). The torque ratio was −0.054 to 0.420 (Figure 5C), which covered the human torque ratio of 0.32 and 0.34 (Figure 4C). This suggested that our exoskeleton could support torque with the same ratio as humans. Current exoskeleton studies have focused on the difference in the adjustment of magnitude and timing of the plantar flexion torque (Slade et al., 2022; Beck et al., 2023; Molinaro et al., 2024), while none of them have investigated the torque ratio of inversion to plantar flexion. In addition, the NM mode can approximate the exoskeleton with the single rope structure, and the results showed that our latBD mode can support more plantar flexion torque and inversion torque than the NM with the same total cable force (Figure 5A, B). Although some exoskeletons can assist the talocrural and subtalar joints separately, suggesting that they have the potential to modulate the torque ratio (Park et al., 2014; Choi et al., 2020; Choi et al., 2022), our proposed method can solve the joint misalignment problem and be more efficient for assisting plantar flexion.

### 4.3 Changes of body stability and human ankle-foot strategy with exoskeleton assistance

**FIGURE 9:**
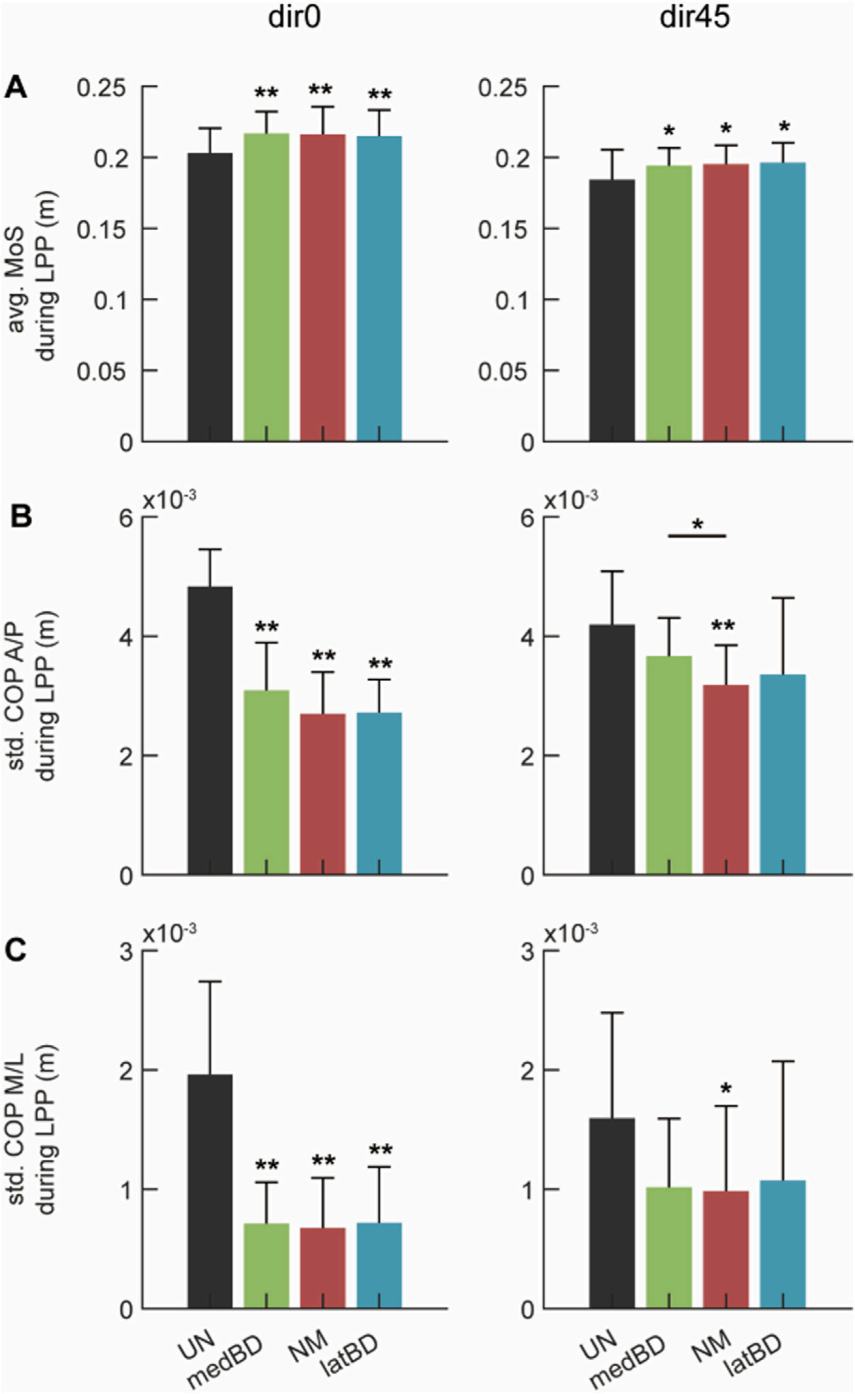
The whole-body stability under dir0 and dir45 conditions: **(A)** the average (avg.) of the Margin of Stability (MoS) during LPP; **(B)** the standard deviation (std.) of the Center of Pressure (CoP) in Anterior/Posterior direction (A/P) during LPP; **(C)** the std. of CoP in Medial/Lateral direction (M/L) during LPP. The black, red, green, and blue represent the UN, NM, medBD, and latBD modes, respectively. The error bars indicate the standard deviation of 7 subjects. The double asterisks above each bar indicate a statistically significant difference compared with UN mode ( n= 7, two-sided paired t -test, p< 0.05). The single asterisk above each bar represents the significant difference compared with NM mode ( n= 7, two-sided paired t -test, p< 0.1). The asterisk with an underline across two bars represents a significant difference between two powered modes ( n= 7, two-sided paired t -test, p< 0.1).

**FIGURE 10:**
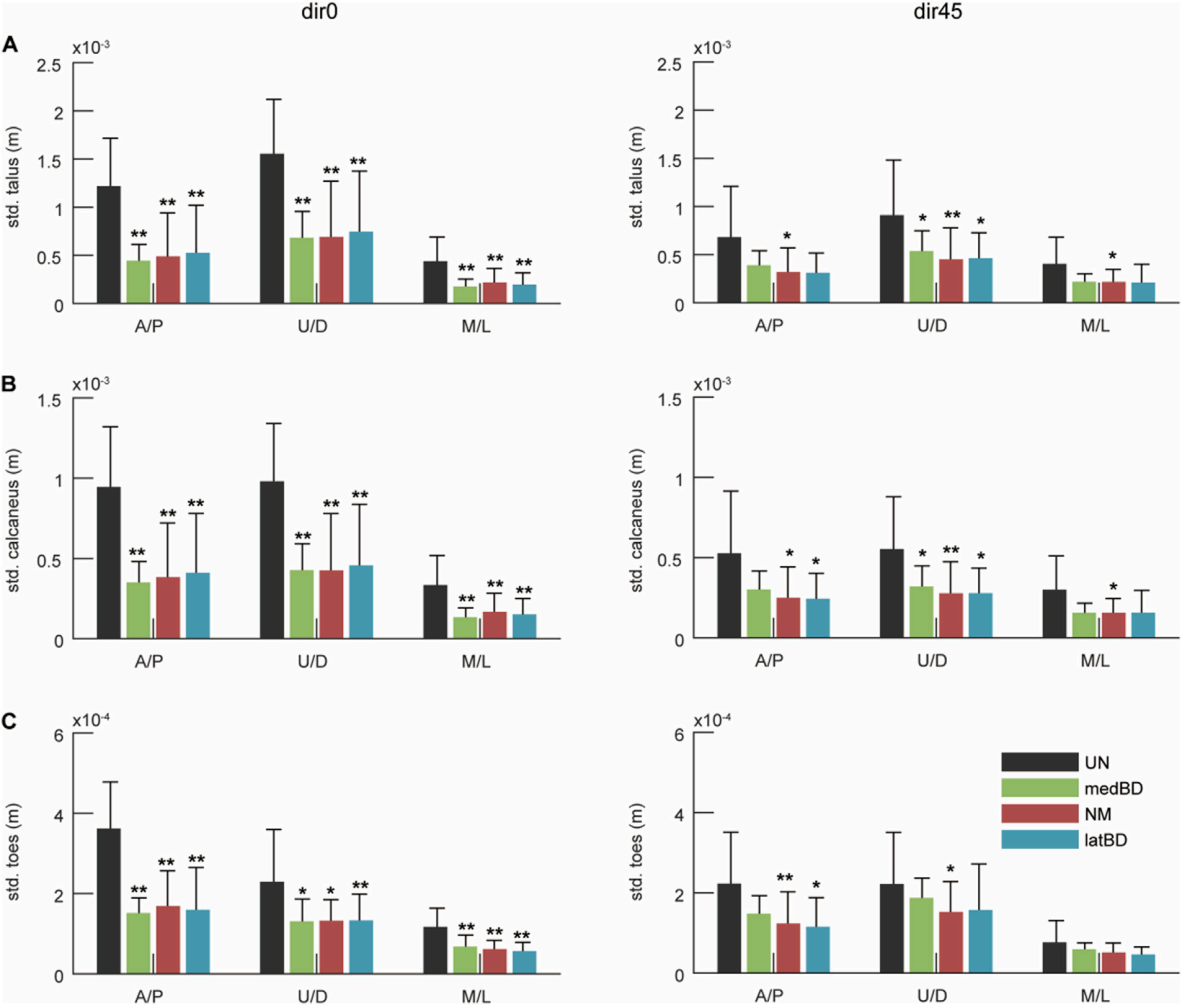
The ankle-foot stability under dir0 and dir45 conditions: the standard deviation (std.) of the Center of Mass (CoM) of **(A)** talus; **(B)** calcaneus; **(C)** toes in A/P, up/down (U/D), and M/L directions during LPP. The black, red, green, and blue represent the UN, NM, medBD, and latBD modes, respectively. The error bars indicate the standard deviation of 7 subjects. The double asterisks above each bar indicate a statistically significant difference compared with UN mode ( n= 7, two-sided paired t -test, p< 0.05). The single asterisk above each bar represents the significant difference compared with NM mode ( n= 7, two-sided paired t -test, p< 0.1).

This study validated that the ankle-foot exoskeleton can assist humans in resisting both forward and left-forward perturbations. For body stability, the MoS increased by about 6.5 % for dir0 and 6 % for dir45 with the exoskeleton compared to the unpowered condition (Figure 9A). The standard deviation of the CoP, another body stability metric, decreased significantly, indicating improved postural stability (Figure 9B). Preliminary evaluation of ankle-foot stability showed that fluctuations in all foot segments were reduced (Figure 10). This study is the first to validate the exoskeleton’s performance under left-forward perturbation.

With similar movement stability, the torque-ratio-adjustable exoskeleton can decrease biological torque of the ankle-foot strategy more effectively than the traditional one. Compared to the unpowered condition, the exoskeleton in NM mode reduced biological plantar flexion torque by 21.6 % and 17.7 % for dir0 and dir45, respectively (Figure 6A), and biological inversion torque by 5.9 % and 8.8 % (Figure 6B). The results under forward perturbation align with previous studies (Asbeck et al., 2015; Emmens et al., 2018; Beck et al., 2023). Among the three powered assistive modes, there was no significant difference in biological plantar flexion torque (Figure 6A). However, the latBD mode reduced biological inversion torque by 10.5 % and 5.4 % more than the NM mode, while the medBD mode performed worse than the NM mode (Figure 6B).

From a muscle perspective, the decrease in biological torque mainly resulted from the reduction in muscle activations of PERL, SOL, and GAS (Figure 7A–D), which formed the muscle synergy related to the “ankle strategy” and was maximally activated from the dir0 to dir45 perturbed direction (Henry et al., 1998; Torres-Oviedo and Ting, 2007). Besides, TA, which contributed to the co-activation of plantar/dorsi flexion, was decreased under dir0 while remaining unchanged under dir45 (Figure 7E). Surprisingly, TP, the main invertor, was increased under dir0 while remaining unchanged under dir45 (Figure 7F). This may be because the activation of the plantar flexor, accompanied by the inversion function, was sharply decreased, leading to inadequate inversion torque under dir0. To compensate for the loss, TP was increased instead, with little plantar flexion function. Furthermore, among the three powered modes, there was no significant difference in the activation of each ankle-foot muscle, except for the TP (Figure 7). The TP activation with latBD was significantly smaller than that of NM, while the medBD was significantly larger. Such a difference was consistent with that of biological inversion torque (Figure 8B).

It was found that the torque ratio absolute difference between the exoskeleton in latBD mode and humans was 0.1 under standard forward perturbation and 0.08 for the left-forward, while the differences for the NM and medBD modes were larger. The absolute differences in torque ratio were relative to the reduction of inversion torque (Figure 8A). The reason was that a similar torque ratio led to less TP compensatory (Figure 8B). The result showed that the latBD had the best performance among the three assistive modes, with a minimum torque ratio absolute difference and a minimum TP activation compensatory. Thus, the hypothesis that the exoskeleton torque ratio should be adjusted to be close to the human torque ratio to get better assistive performance was validated.

## 5 Conclusion

This study proved that the human natural torque ratio of inversion to plantar flexion was modulated according to the perturbation direction. Based on the regulation demand, we developed an ankle-foot exoskeleton that can adjust the torque ratio delivered to the human body by controlling the forces on two cross-arranged cables. The adjustable range can cover the human ratio under current structural size constraints. The assistive performance validation experiment demonstrated that the torque-ratio-adjustable exoskeleton can support humans in resisting forward-type perturbations. As far as we know, this study was the first to investigate the performance of the exoskeleton under perturbations outside the sagittal plane. Furthermore, it was found that the biological inversion torque was reduced more with the smaller torque-ratio difference between the exoskeleton and humans, which indicated that the torque ratio of the exoskeleton should be carefully modulated according to that of humans under diverse environmental conditions for better assistive performance.

## Data Availability

The raw data supporting the conclusions of this article will be made available by the authors, without undue reservation.
